# Somatic Cell Nuclear Transfer Followed by CRIPSR/Cas9 Microinjection Results in Highly Efficient Genome Editing in Cloned Pigs

**DOI:** 10.3390/ijms17122031

**Published:** 2016-12-03

**Authors:** Timothy P. Sheets, Chi-Hun Park, Ki-Eun Park, Anne Powell, David M. Donovan, Bhanu P. Telugu

**Affiliations:** 1 Department of Animal and Avian Sciences, University of Maryland, College Park, MD 20742, USA; tsheets@umd.edu (T.P.S.); chpark@umd.edu (C.-H.P.); kepark@umd.edu (K.-E.P.); 2 Animal Bioscience and Biotechnology Laboratory, USDA-ARS, Beltsville, MD 20705, USA; anne.powell@ars.usda.gov (A.P.); david.donovan@ars.usda.gov (D.M.D.); 3 Renovate Biosciences Inc., Reisterstown, MD 21136, USA

**Keywords:** CRISPR/Cas, SCNT, pig, knockout, microinjection

## Abstract

The domestic pig is an ideal “dual purpose” animal model for agricultural and biomedical research. With the availability of genome editing tools such as clustered regularly interspaced short palindromic repeat (CRISPR) and associated nuclease Cas9 (CRISPR/Cas9), it is now possible to perform site-specific alterations with relative ease, and will likely help realize the potential of this valuable model. In this article, we investigated for the first time a combination of somatic cell nuclear transfer (SCNT) and direct injection of CRISPR/Cas ribonucleoprotein complex targeting *GRB10* into the reconstituted oocytes to generate *GRB10* ablated Ossabaw fetuses. This strategy resulted in highly efficient (100%) generation of biallelic modifications in cloned fetuses. By combining SCNT with CRISPR/Cas9 microinjection, genome edited animals can now be produced without the need to manage a founder herd, while simultaneously eliminating the need for laborious in vitro culture and screening. Our approach utilizes standard cloning techniques while simultaneously performing genome editing in the cloned zygotes of a large animal model for agriculture and biomedical applications.

## 1. Introduction

The pig is an ideal model for agricultural and biomedical research. Even though, the rodent models have great utility in translational medicine [1,2,3,4,5], the small size, short life span, greater evolutionary distance, dissimilarities in physiology, and an increasing number of drugs failing at an advanced stage of clinical development in these models have highlighted the need for alternative animal models. In this regard, animal models such as pigs that can serve as a bridge model between rodents and expensive primate models are being increasingly coveted [3]. Additionally, research in pigs will have added value to agriculture and veterinary medicine. The development and successful application of modern gene editing technologies such as clustered regularly interspaced short palindromic repeat and associated nuclease Cas9 (CRISPR/Cas9) in the pig model, now offer a significantly improved outlook towards future translational and dual purpose research studies.

**Figure 1:**
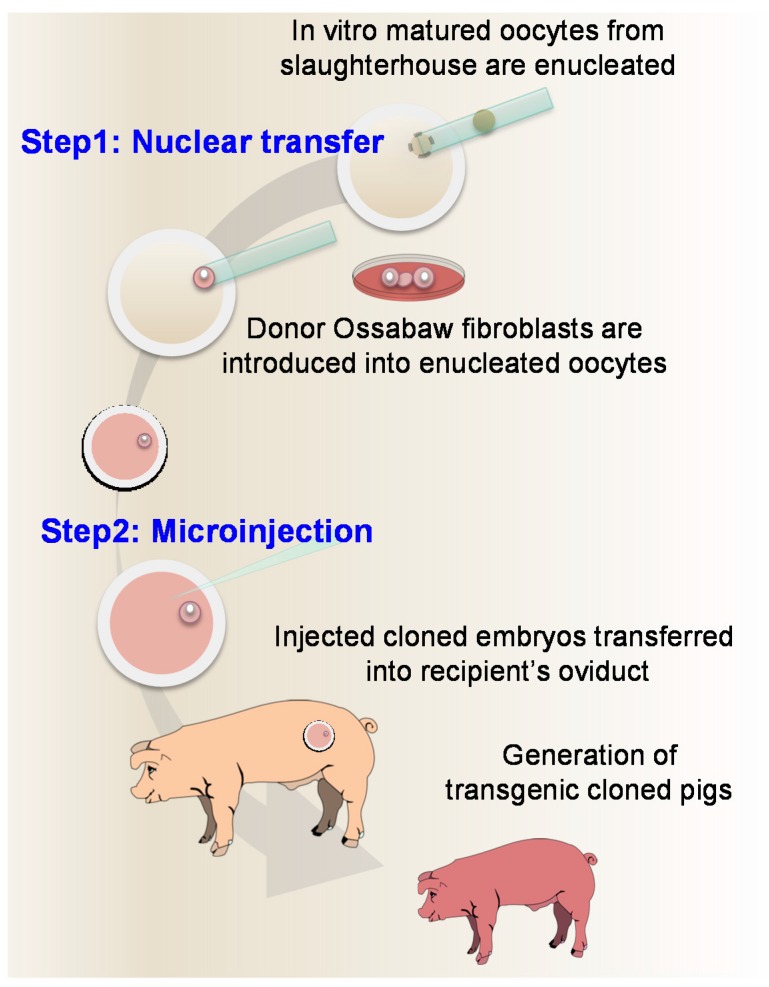
Schematic outlining the strategy for editing cloned zygotes.

Among the different editors, including the ZFNs (zinc finger nucleases) and TALENS (TAL effector nuclease), the CRISPR/Cas9 system has found greater application due to the ease in design, preparation, and delivery. This technology requires the use of a short stretch of RNA binding sequence to the Cas9 nuclease alongside a 20-mer sequence specific to the genomic target (guide RNA) sequence, which together can be prepared as a chimeric single guide RNA (sgRNA). The CRISPR reagents can be delivered as expression plasmids, in vitro transcribed RNA, or the commercially available Cas9 protein pre-complexed with sgRNA. When introduced into somatic cells or embryos, these can introduce double strand breaks (DSB) and facilitate targeted gene modifications. In domestic pigs, the conventional means for generating genetically engineered animals is somatic cell nuclear transfer (SCNT) or cloning, where somatic cells (typically fetal fibroblasts) are modified to introduce the intended genetic modification and then used as nuclear donors for SCNT. However, the efficiency of somatic cell gene targeting is typically low and requires a laborious and time-consuming screening process. Additionally, extended culture and screening in vitro can result in significant reductions in cell viability, ultimately affecting overall cloning efficiency. In this regard, microinjection of CRISPR reagents directly into the embryos is a straightforward method with high efficiencies [6]. That said, direct injections into the embryo requires a founder herd of male and female animals, synchronization of donor animals, breeding, and surgical recovery of embryos necessitating significant investment of resources and infrastructure. On the other hand, the use of in vitro fertilized zygotes as a source is hampered by high incidence of polyspermy [7]. Given these limitations, the main goal of this study was to combine the advantages of SCNT requiring only the somatic cells and slaughter-house derived oocytes with direct injection of CRISPR reagents into embryos for the generation of targeted mutations in the desired genetic background. As a proof of principle, we tested the feasibility of ablating *GRB10* gene in Ossabaw background (Figure 1).

Growth-hormone receptor binding protein-10 (GRB10) is an adaptor protein so called because of the ability to bind but not activate receptors. GRB10 has been identified as a negative regulator of insulin signaling via its binding and direct repression of insulin-like growth factor 1/2 and the insulin receptor [8,9,10,11,12,13,14]. Studies from knockout mice have identified an overgrowth phenotype most notable in utero in placental mass [15,16], and postnatally in the liver [15], muscle [10,17,18], adipose depot [10], islet cell mass [19], and overall body mass [10,17,18], confirming the role of GRB10 as a repressor of insulin signaling. Consistent with the role as growth repressor, overexpression of *GRB10* resulted in fetal growth restriction in utero with a correlative decrease in organ and body mass [20]. However, discrepancies in imprinting status have been reported between mouse and humans, and functional interpretation of the function of GRB10 in non-rodent model has not been investigated yet [21]. The investigation of role in insulin resistance and obesity in Ossabaw pigs that have a naturally existing thrifty phenotype, and can develop obesity when fed an obesogenic diet is anticipated to be a worthwhile investigation. As a necessary first step, in this article, the feasibility of generating targeted modifications in SCNT embryos derived from Ossabaw fibroblasts and direct injection of CRISPR ribonucleoproteins ablating *GRB10* in the resulting fetuses has been investigated.

## 2. Results and Discussion

In the current study, a combined approach of SCNT followed by microinjection of CRISPR/Cas9 reagents to generate targeted gene knockout in Ossabaw cloned pigs was investigated (Figure 1). A typical cloning strategy involves plasmid-based expression of CRISPR/Cas9 or CRISPR ribonucleoproteins to target the genome of somatic cells in vitro, followed by antibiotic selection or single cell sorting using a co-transfected fluorescent reporter [22]. Sustained plasmid expression has been shown to increase off-target mutations [23,24]. Clonal cells are then laboriously screened for desired genotype and subsequently used as nuclear donor for SCNT. Microinjection of either guide RNA with Cas9 mRNA or sgRNA/Cas9 protein complexes directly into cytoplasm of zygotes has been shown to be an effective means for generating gene-edited animals [6]. We have improved upon SCNT and genome editing by combining the two techniques in the same embryos thus eliminating the need for pre-screening or selection of mutational events in somatic cells and/or microinjected embryos.

**Figure 2:**
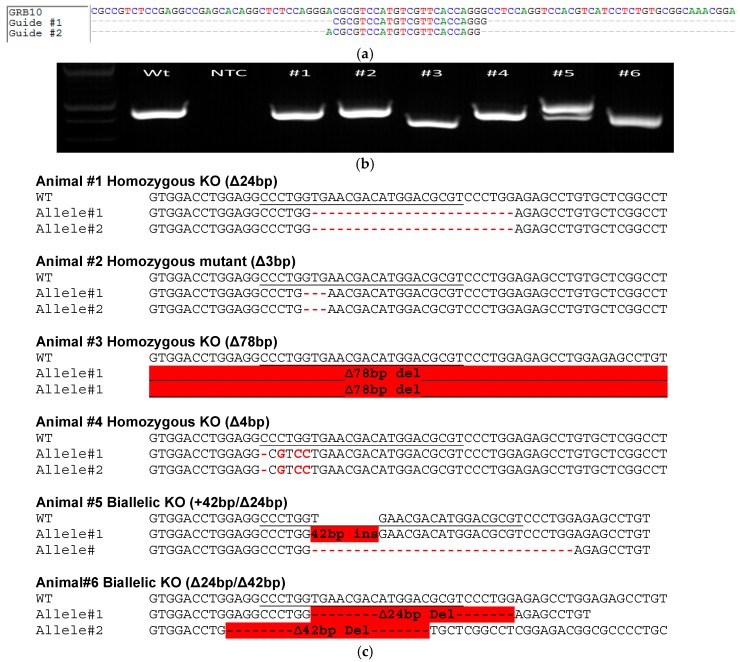
Genotyping of *GRB10* in day 60 cloned Ossabaw fetuses. (**a**) Multiple sequence alignment showing the two guide sequences relative the wild type sequence in Exon 4 of *GRB10* gene; (**b**) Agarose gel electrophoresis image of *GRB10* amplicons from fetuses (numbered 1–6) showing noticeable shift in size in Fetus #3 and #6 and two differently targeted alleles in Fetus #5. Amplicon from wildtype (WT) animal is shown as a reference; (**c**) Genomic DNA was extracted from ear and tail biopsies, cloned into PCR2.1 vector, and 10 representative bacterial clones from each sample and from each fetus was sequenced by Sanger sequencing, confirming biallelic edits in all six fetuses.

The first step was to determine whether microinjection of cloned zygotes following extensive manipulation of oocyte such as enucleation, somatic cell injection and electrofusion will compromise developmental competence. To assess this, SCNT was carried out using fibroblasts derived from an Ossabaw (Day 39) pig fetus. Following electroactivation and 4 h incubation with 6-dimethylaminopurine (6-DMAP), the reconstituted cloned zygotes were microinjected with a combination of two sgRNAs targeting exon 4 of *GRB10* (Figure 2a) each pre-complexed with Cas9 protein. The two guides that differ in sequence by one base pair were used in order to increase the likelihood of a targeted DSB at the target site (Figure 2a). Bui et al., showed that following somatic cell injection into an oocyte, a metaphase-like stage reaches its peak after 2 h and a pronuclear structure is formed within 6 h [25]. In this study, the timing of microinjection into cloned zygotes, i.e., after 4 h of 6-DMAP treatment was the earliest time point when the SCNT procedure is completed, and secondly, this is a time when pronuclear-like structure is yet to be formed in cloned zygotes. Therefore, this time was empirically chosen for balancing cloning and gene targeting efficiencies. As shown in Table 1, the process of microinjection did not affect early cleavage of cloned zygotes, but showed relatively lower blastocyst rates compared to uninjected controls. However, no statistically significant difference was found in the overall development rates between these two groups (Student’s *t*-test: *p* = 0.37 and *p* = 0.08, respectively). Because, this process resulted in a reasonable development rate of cloned zygotes following microinjection, we performed embryo transfers into synchronized surrogate animals to test whether the microinjected embryos can establish a successful pregnancy. A total of 115 and 76 embryos were transferred into two surrogate gilts, respectively, which resulted in one successful pregnancy (Table 2). Six fetuses were recovered from the pregnant animal at embryonic day 60 (E60). The overall efficiency after SCNT followed by CRISPR/Cas9 ribonucleoprotein complex microinjection was 3.1%. Screening of genomic DNA from ear and tail samples using *GRB10* specific primers (Table 3; Figure 2b) revealed that all fetuses carried biallelic edits for the *GRB10* gene (6/6, 100%, Figure 2c). No evidence of mosaicism was observed (e.g., more than 2 alleles) from cytoplasmic microinjection of ribonucleoprotein complexes [22].The shorter bio-availability of ribonucleoproteins compared to plasmid-based expression and mRNA alone was expected to limit the length of exposure to the genome, reducing mutational events past initial DNA replication. This in turn increases the incidence of biallelic modifications. Similar observations have been observed for CRISPR/Cas9 injections in porcine embryos [6,23,26].

In summary, the data indicates that genome editing by direct cytoplasmic injection of CRISPR/Cas9 ribonucleoprotein complexes into zygotes produced by SCNT (with commercially available oocytes and donor nuclei from specialty breed fibroblasts (e.g., Ossabaw)) enables rapid and efficient gene targeting in specialty pig breeds (Table 2). Using this combined technique, the need for donor embryos from specialty herds, cell culture screening, and incidence of mosaicism in the resultant progeny has been eliminated. We conclude that this approach is a cost- and resource-saving means for generating biallelic gene edits in cloned pigs with high efficiency.

## 3. Experimental Section

All chemicals were obtained from Sigma Chemical Company (St. Louis, MO, USA) unless stated otherwise. All animal work was performed under approved protocols from ARS, USDA, Beltsville, MD, Animal care and use committee.

### 3.1. Production of sgRNA

Targeting guide RNAs were designed based on the software available from MIT (http://www.genome-engineering.org/crispr/). Two complementary sgRNA oligo DNAs (24 nucleotides in length) were synthesized by IDT (IDT DNA technologies), annealed to form double-strand DNA and cloned into a Bsa1 restriction enzyme digested in-house T7 promoter driven vector. The cloned fragments were DNA sequenced to confirm their fidelity and in vitro transcribed using MEGAshortscript T7 kit (Life Technologies, Carlsbad, CA, USA) to generate chimeric sgRNAs. In vitro transcribed sgRNA was purified with MEGAclear kit (Life Technologies) and eluted in RNase-free water for embryo injections.

### 3.2. Somatic Cell Nuclear Transfer

All animal work was performed as per the approved guidelines of Beltsville ARS, Institutional Animal Care and Use Committee (AUPF-3). SCNT was performed as described in previous study [27]. Cumulus-oocyte complexes (COCs) were purchased from a commercial supplier (De Soto Biosciences, Seymour, TN, USA). Briefly, matured oocytes were enucleated by aspirating the polar body and MII chromosomes with an enucleation pipette (Humagen, Charlottesville, VA, USA). After enucleation, a donor cell was introduced into the perivitelline space of an enucleated oocyte. Fusion of injected oocytes was induced by DC pulse (2.0 kV/cm for 30 µs using a BTX-Cell Manipulator 2001 (BTX)). After fusion, the reconstructed oocytes were activated by an electric pulse (1.0kV/cm for 60 μs), followed by 4 hof incubation in Porcine Zygote Medium-3 (PZM3) medium containing 2 mM 6-DMAP.

### 3.3. Microinjection of Embryos

After 4 h incubation in 6-DMAP, cloned zygotes were microinjected with a mixture of Cas9 protein and sgRNA mixture using a FemtoJet microinjector (Eppendorf, Hamburg, Germany). Briefly, 20 pL of precomplexed Cas9 protein (25 ng/μL) and both *GRB10* sgRNAs (12.5 ng/μL each) in nuclease free water was injected with hand-made pipette pulled on Flaming Brown Micropipette Puller (Sutter Instrument Co., Novato, CA, USA). Embryos that did not receive injections were used as controls. These embryos were cultured to blastocyst stage in PZM3 medium for 144 h at 38.5 °C, 5% CO_2_, 5% O_2_ and 100% humidity for screening.

### 3.4. Embryo Transfer

After microinjection, the injected SCNT embryos were in vitro cultured for one-two day and approximately 96 (95.5) embryos at the 2–8 cell stage were surgically transferred into the oviducts of synchronized gilts on the first day of standing estrus. Pregnancies were confirmed by ultrasound on day 30 following transfer, fetuses were harvested from pregnant day 60 euthanized sow. Muscle tissues from 6 fetuses was trypsin digested, cultured and frozen in liquid nitrogen.

### 3.5. Genotyping of Embryos, Fetuses and Edited Animals

In vitro cultured single blastocysts cultured for 144 h were washed three times with phosphate buffered saline-polyvinyl alcohol (pH 7.4) medium (PBS-PVA) and transferred into 9 µL of blastocyst lysis buffer (50 mM KCl, 1.5 mM MgCl_2_, 10 mM Tris pH 8.0, 0.5% NP-40, 0.5% Tween-20 and 100 µg/mL proteinase K) and incubated for 1 h at 65 °C. The digestion was terminated by heating the mixture at 95 °C for 10 min, and 2 µL of supernatant was used as a Polymerase chain reaction (PCR) template. Tissue biopsies (ear notch and tail dock) from fetuses were digested in a tissue lysis buffer (50 mM Tris pH 8.0, 0.1 M NaCl, 20 mM DTA, 1% SDS, 50 µg/mL RNase A, 100 µg/mL proteinase K) overnight at 55 °C. Following overnight digest, the genomic DNA of the sample was extracted from the tissue lysate using phenol-chloroform, and recovered by resuspension in 100 µL of 10 mM Tris-HCl, pH 7.4 buffer following ethanol precipitation. Purified genomic DNA was amplified using PCR, cloned into PCR2.1 vectors (Life Technologies) and transformed into *Ε. coli* DH5-α maximum competent cell (Life Technologies). Five to ten colonies were picked, cultured, plasmid DNA extracted and sequenced (Macrogen, Rockville, MD, USA). Sequences were aligned by Bio-Edit software (Ibis Biosciences, Carlsbad, CA, USA) for comparison with wild-type alleles.

## Figures and Tables

**Table 1:** Developmental competence of reconstructed cloned embryos followed by single guide RNA (sgRNA) microinjection.

Groups		No. Cultured	No. Cleaved (%)	No. Blastocysts (%)
SCNT	Control	60	54 (90.0 ± 3.3)	33 (61.5 ± 11.6)
SCNT	Injected	77	71 (91.8 ± 3.6)	25 (34.5 ± 4.5)

Data on developmental rates was analyzed using unpaired student’s *t*-tests. There was no statistically significant difference between groups in cleavage and blastocyst rates (*p* = 0.37 and *p* = 0.08, respectively). SCNT: somatic cell nuclear transfer.

**Table 2:** Generation of clustered regularly interspaced short palindromic repeat and associated nuclease Cas9 (CRISPR/Cas9)-mediated *GRB10* targeted cloned fetuses.

No. Embryos Transferred	No. of Recipients	No. Pregnant (%)	No. of Fetuses and (CE %) *	No. of Bi-Allelic Edited (%)
191	2	1/2 (50)	6 (3.1)	6/6 (100)

* Cloning efficiencies (CE) were calculated as the percentage of the number of fetuses relative to the number of embryos transferred to recipients.

**Table 3:** Guide Oligos and Genotyping Primers used in the manuscript.

Oligo/Primer	Sequence	Length
Guide 1 Forward	TAGGCGCGTCCATGTCGTTCACCA	24nt
Guide 1 Reverse	AAACTGGTGAACGACATGGACGCG	24nt
Guide 2 Forward	TAGGACGCGTCCATGTCGTTCACC	24nt
Guide 2 Reverse	AAACGGTGAACGACATGGACGCGT	24nt
*GRB10* Forward	GATGTGTGCTGTGGAACCGA	20nt
*GRB10* Reverse	CCCTTAGCCCACTTACTCCAGAC	23nt
